# Binding energies and the entry route of palmitic acid and palmitoylcarnitine into myoglobin

**DOI:** 10.1016/j.dib.2018.10.118

**Published:** 2018-10-27

**Authors:** Sree V. Chintapalli, Andriy Anishkin, Sean H. Adams

**Affiliations:** a Arkansas Children׳s Nutrition Center and Department of Pediatrics, University of Arkansas for Medical Sciences, Little Rock, USA; b Department of Biology, University of Maryland, College Park, USA

## Abstract

The interaction of lipids (entry mechanism) with respect to both oxy- and deoxy-myoglobin was explored using unrestrained Molecular Dynamics simulations. The results indicated a spontaneous entry of both palmitic and palmitoylcarnitine molecules into the oxy-Mb structure at the main binding site, whereas in deoxy-Mb, both the lipid ligands move away from the protein surface. For the alternative binding locations, entry of the ligands was independent of the oxygenation state. Presented here are the tables with the myoglobin binding energies for palmitic acid and palmitoylcarnitine estimated using Alchemical Free Energy Perturbation approach for the key structures obtained in unrestrained Molecular Dynamics simulations. These data are referenced in the original article “Exploring the entry route of palmitic acid and palmitoylcarnitine into myoglobin”, reference number YABBI7787.

**Specifications table**

**Table:** 

Subject area	*Biochemistry, Physiology, Bioinformatics*
More specific subject area	*Molecular Dynamic Simulations*
Type of data	*Binding Energy estimations*
How data was acquired	*MD Simulations*
Data format	*Filtered, analyzed*
Experimental factors	*The starting conformations of the mouse myoglobin were created using homology modeling based on the published horse myoglobin structures*[1], [2].
Experimental features	*Molecular Dynamics simulations in all-atom explicit medium using CHARMM36 force field*
Data source location	*Simulated data were obtained at Arkansas Children׳s Nutrition Center, University of Arkansas for Medical Sciences, Little Rock, USA*
Data accessibility	*Atomic coordinate and any additional information is available upon request.*
Related research article	“Exploring the entry route of palmitic acid and palmitoylcarnitine into myoglobin”, reference number YABBI7787

**Value of the data**

• Describes myoglobin׳s new role in lipid transport along with oxygen carrier/storage.
• Addresses lipid release from myoglobin on deoxygenation.
• Suggests potential impact of the bound lipids on oxygen release from myoglobin.
• The results open new doors to study other globin members and their involvement with lipid metabolism.

## 1 Data

The resented data illustrate the results of Molecular Dynamics simulations of the lipids (palmitic acid and palmitoylcarnitine) interacting with mouse myoglobin in oxygenated and deoxygenated states. Here, we provide energy estimates for the binding using Free Energy Perturbation approach and visualization of multiple interaction events.

## 2 Experimental design, materials, and methods

Unrestrained Molecular Dynamics simulations were performed using NAMD software [3], CHARMM36 force field, TIPT3P water model with rigid bonds, PME approximation for long-range electrostatics, 12 Å cutoff for direct non-bonded interactions. See details in the main text.

### 2.1 Free energy perturbation (FEP) estimates for the bound and unbound conformations of PLM and PLC with Mb

FEP estimations were performed using FEP module of NAMD [3]. The starting conformations for both palmitate (PLM) and palmitoylcarnitine (PLC) were taken at the end of several MD simulations representative of the major binding locations – the main pocket, side-attached, A1, A2, and an unbound (diffuse) location. For each of the locations, we have performed an “Alchemical” FEP with the molecule gradually disappearing in the starting location and appearing in the bulk water, followed by the reverse process (disappearance from the bulk and re-appearance in the starting conformation). The target bulk water location was chosen at the region most remote from all the periodic copies of the protein. To prevent protein drift during the FEP simulations, the alpha-carbons of 19 protein residues positioned at the most remote corners of Mb were harmonically restrained to the starting positions with a very soft constant of 0.01 kcal/mol/Å. We have applied the same soft harmonic restraint to a single carbon atom (C8) of a target lipid molecule in the bulk to prevent diffusion and contact with the protein, while still allowing rotation and conformational change. For the lipid molecules bound to Mb, we have applied 0.01 kcal/mol/Å restraint to all heavy atoms to avoid drift into non-natural locations in the protein on re-appearance, which otherwise was observed for an unrestrained lipid molecule. We should note that with the restraints being so soft, the entropic effect on the available range of conformations was negligible, with lipid molecules exploring essentially the same conformational space allowed by the surrounding as unrestrained molecules – only the significant diffusion was prevented. It is reflected in the estimate of the free energy cost of the transfer from the diffuse (unbound) starting location to the bulk solution, which was less than 1 kcal/mol (see the table). Each of the forward and backward FEP transformations were performed over 2 ns with 20 intermediate windows (100 ps per window), with the coupling parameter changing linearly between 0 and 1 in 0.05 increments. The first 10 ps of each window were allowed for system equilibration, followed by 90 ns of FEP data collection. The rest of the simulation conditions were identical to those in our unrestrained MD simulations described in the main text. The output from the forward and backward transformations was integrated using SOS estimator for FEP analysis plugin in VMD [4], [5]. The results of the free energy estimations are presented in Table 1 (Table 2).

**Table 1:** Distances characterizing the conformational change in the main site on binding of PLM and PLC to Oxy-Mb. The distances were measured independently for each simulation, for the conformation aligned by the protein backbone and time-averaged over the last 5 ns. The distance measurements were averaged among all the simulations fitting into one of the four types: 1) with PLM bound to the main site, 2) PLC bound to the main site, 3) PLM not bound to the main site (i.e. bound elsewhere or in solution), and 4) PLC not bound to the main site. The distances were calculated for pairs of representative atoms as indicated in the table.

**Dimension type →**	**Crevice diagonal**	**Distance across the heme (H) plane**	**Distance along the heme (H) plane**	**Entrance to the crevice**	**Distance to the inner heme (H) side (along the H plane)**	**Distance to the inner heme (H) side (across H plane)**	**Distance to the outer heme (H) side (along the H plane)**	**Distance to the outer heme (H) side (across the H plane)**
**Models ↓ Atoms→**	CA(L29)-CA(H93)	CA(H64)-CA(H93)	CA(A71)-CA(I107)	CA(F43)-CA(H97)	CA(I107)-CHC (H)	CA(L29)-CHC (H)	CA(I107)-CHA(H)	CA(D60)-CHA(H)
**OXYMB+PLM (main site)**	16.6±0.8	15.3±0.4	13.6±0.5	11±2.1	6.8±0.3	9.9±0.6	13.1±0.4	14.8±1.0
**OXYMB+PLC (main site)**	16.8±0.7	15.4±0.2	13.5±0.6	12.3±1.6	6.7±0.4	10.2±0.5	13.0±0.4	15.1±0.4
**OXYMB+PLM (not main site)**	15.7±0.3	14.5±0.3	13.3±0.9	9.2±1.6	6.4±0.2	9.5±0.4	12.3±0.5	13.1±0.6
**OXYMB+PLC (not main site)**	15.7±0.2	14.6±0.2	13.2±1.0	9.2±1.1	6.5±0.1	9.3±0.2	12.6±0.2	12.9±0.8

**Table 2:** FEP estimates of the five different major binding locations namely the main pocket, side-attached, A1, A2, and an unbound (diffuse) location. The free energy estimations (ΔG, kcal/mol) for both PLM and PLC are listed.

**Binding location**	**ΔG, kcal/mol**	
	**PLM**	**PLC**
**Main pocket, Oxy-Mb**	11.5±0.5	18.3±0.6
**Side attached, Oxy-Mb**	4.4±0.4	6.6±0.6
**Diffuse, Deoxy-Mb**	−0.9±0.4	0.5±0.5
**A1, Oxy-Mb**	20.4±0.5	19.6±0.5
**A2, Oxy-Mb**	16.5±0.6	8.4±0.5
