# Supplementary material for: Binding energies and the entry route of palmitic acid and palmitoylcarnitine into myoglobin
Source: Data Brief. 2018 Oct 27;21:1106–10. doi: 10.1016/j.dib.2018.10.118 (PMC6231043; doi:10.1016/j.dib.2018.10.118)
Supplement: Supplementary file 1 — Supplementary material [file mmc1.docx]

**Conflict of Interest:** The authors declare that they have no conflict of interests with the contents of this article.
